# Supplementary material for: Insights into Adaptive Regulation of the Leaf-Petiole System: Strategies for Survival of Water Lily Plants under Salt Stress
Source: Int J Mol Sci. 2023 Mar 15;24(6):5605. doi: 10.3390/ijms24065605 (PMC10058412; doi:10.3390/ijms24065605)
Supplement: Supplementary file 1 [file ijms-24-05605-s001.zip › ijms-2180570-supplementary.pdf]

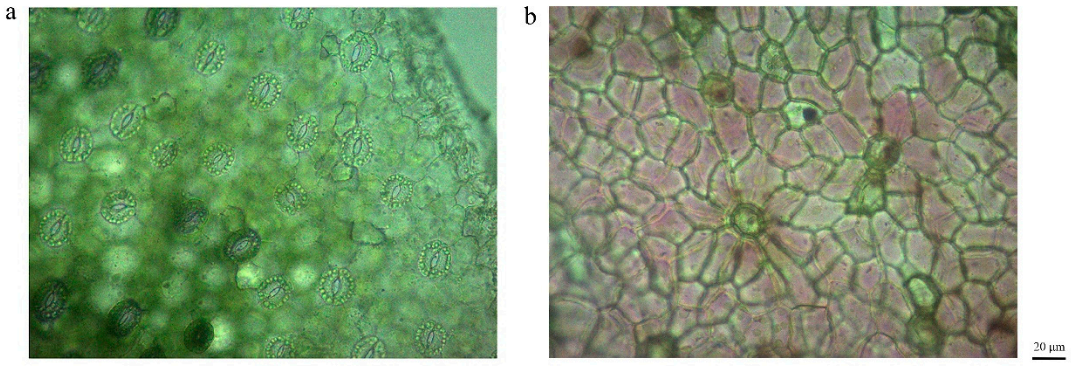

Figure S1 Observation of water lily leaves with an optical microscope. (a) Image of the upper epidermis of water lily leaves. **(b)** Image of the lower epidermis of water lily leaves.

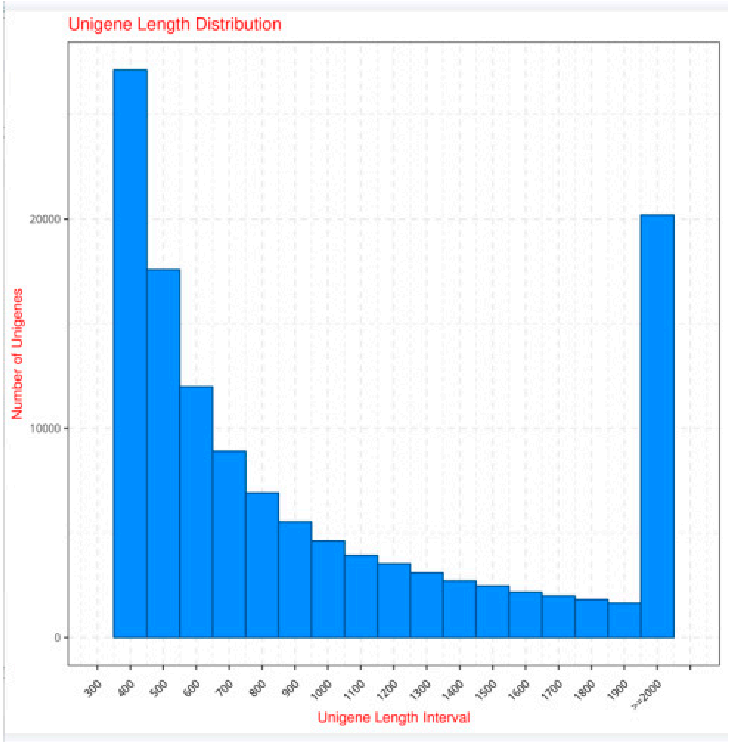

Figure S2 Histogram of unigene length distributions. The x-axis indicates sequence sizes from 300 bp to over 2000 bp. The y-axis indicates the number of unigenes for every given size.

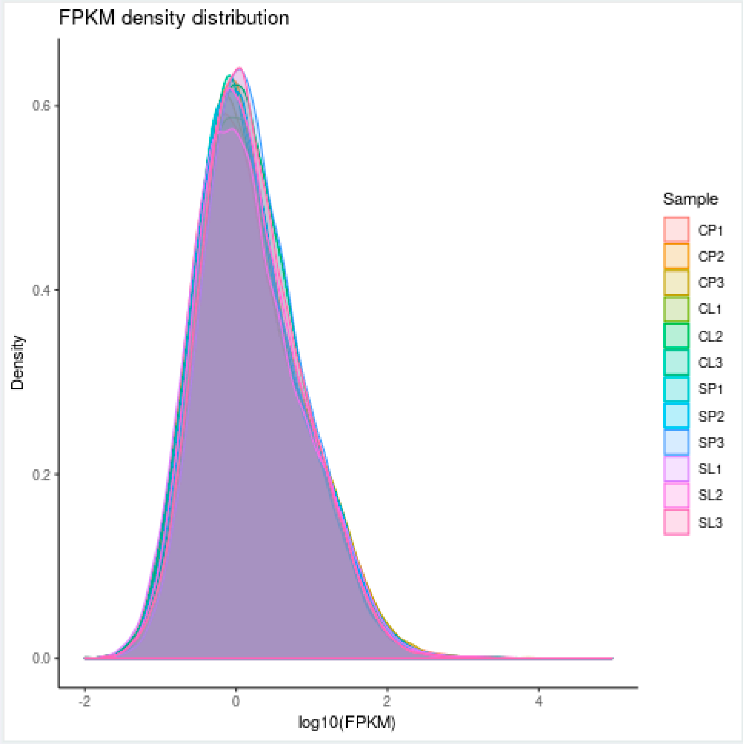

Figure S3 The FPKM distribution of different tissue samples. Density distributions of FPKM based on $\log_{10}$ (FPKM) showed the gene expression level over tissues. The x axis is $\log_{10}$ (FPKM), and the y axis is the density of the gene. CP, petioles of control plants; CL, leaves of control plants; SP, petioles of salt-treated plants; SL, leaves of salt-treated plants.

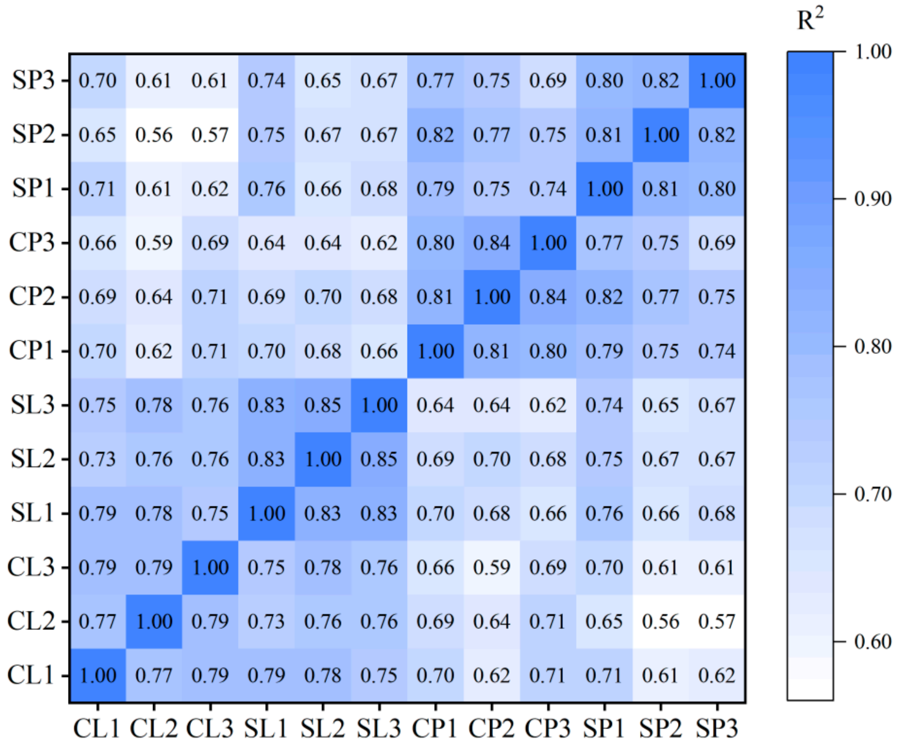

Figure S4 Pearson correlation between samples. R^2 represents Pearson correlation coefficient. CP, petioles of control plants; CL, leaves of control plants; SP, petioles of salt-treated plants; SL, leaves of salt-treated plants.

Table S1 Summary of the mapping information

Sample name	Total reads	Total mapped	Percentage
CP1	39,916,042	27,789,714	69.62%
CP2	45,129,198	31,552,978	69.92%
CP3	42,557,834	29,586,096	69.52%
CL1	39,737,182	27,383,066	68.91%
CL2	47,406,414	31,627,328	66.72%
CL3	41,044,468	28,240,426	68.80%
SP1	42,928,630	29,630,696	69.02%
SP2	46,198,250	31,910,974	69.07%
SP3	42,340,004	28,944,202	68.36%
SL1	41,694,494	28,841,444	69.17%
SL2	40,239,300	28,025,498	69.65%
SL3	46,197,784	32,188,306	69.67%

Table S2 Annotation statistics of Nr, Nt, Pfam, KOG/COG, Swiss-Prot, KO and GO database

	Number of Unigenes	Percentage (%)
Annotated in NR	59,928	43.97
Annotated in NT	24,031	17.63
Annotated in KO	18,508	13.58
Annotated in SwissProt	42,391	31.1
Annotated in PFAM	39,897	29.27
Annotated in GO	39,888	29.26
Annotated in KOG	8533	6.26
Annotated in all Databases	3946	2.89
Annotated in at least one Database	73,410	53.86
Total Unigenes	136,306	
